# Rescue of HSP70 in Spinal Neurons Alleviates Opioids-Induced Hyperalgesia via the Suppression of Endoplasmic Reticulum Stress in Rodents

**DOI:** 10.3389/fcell.2020.00269

**Published:** 2020-05-12

**Authors:** Tong-Tong Lin, Jie Qu, Chao-Yu Wang, Xing Yang, Fan Hu, Liang Hu, Xue-Feng Wu, Chun-Yi Jiang, Wen-Tao Liu, Yuan Han

**Affiliations:** ^1^Neuroprotective Drug Discovery Key Laboratory, Department of Pharmacology, Nanjing Medical University, Nanjing, China; ^2^State Key Laboratory of Pharmaceutical Biotechnology, Nanjing University, Nanjing, China; ^3^Institute of Translational Medicine, Nanjing Medical University, Nanjing, China; ^4^Jiangsu Province Key Laboratory of Anesthesiology, Xuzhou Medical University, Xuzhou, China

**Keywords:** opioids-induced hyperalgesia, endoplasmic reticulum stress, unfolded protein response, HSP70, PKA, NR1

## Abstract

A major unresolved issue in treating pain is the paradoxical hyperalgesia produced by the gold-standard analgesic morphine and other opioids. Endoplasmic reticulum (ER) stress has been shown to contribute to neuropathic or inflammatory pain, but its roles in opioids-induced hyperalgesia (OIH) are elusive. Here, we provide the first direct evidence that ER stress is a significant driver of OIH. GRP78, the ER stress marker, is markedly upregulated in neurons in the spinal cord after chronic morphine treatment. At the same time, morphine induces the activation of three arms of unfolded protein response (UPR): inositol-requiring enzyme 1α/X-box binding protein 1 (IRE1α/XBP1), protein kinase RNA-like ER kinase/eukaryotic initiation factor 2 subunit alpha (PERK/eIF2α), and activating transcription factor 6 (ATF6). Notably, we found that inhibition on either IRE1α/XBP1 or ATF6, but not on PERK/eIF2α could attenuate the development of OIH. Consequently, ER stress induced by morphine enhances PKA-mediated phosphorylation of NMDA receptor subunit 1(NR1) and leads to OIH. We further showed that heat shock protein 70 (HSP70), a molecular chaperone involved in protein folding in ER, is heavily released from spinal neurons after morphine treatment upon the control of K_ATP_ channel. Glibenclamide, a classic K_ATP_ channel blocker that inhibits the efflux of HSP70 from cytoplasm to extracellular environment, or HSP70 overexpression in neurons, could markedly suppress morphine-induced ER stress and hyperalgesia. Taken together, our findings uncover the induction process and the central role of ER stress in the development of OIH and support a novel strategy for anti-OIH treatment.

## Introduction

Opioids, such as morphine, are indispensable in the treatment of severe pain. However, use of these drugs is plagued by two major unresolved problems: hyperalgesia and tolerance. Tolerance is manifested clinically by the need for increasing opioid dosages over time to maintain the same level of pain relief; this increased need is not explained by disease progression. The mechanism of morphine tolerance is complicated and involves many aspects, such as receptors (e.g., decreased receptor activation, desensitization, or opioid receptor down-regulation), ion channels (the decreased influx of Ca^2+^ and the increased efflux of K^+^), and neuroinflammation (activation of microglia cell) (Williams et al., 2013). Hyperalgesia is a paradoxical sensitization process in which opioids instead cause pain hypersensitivity (Bekhit, 2010; Lee et al., 2011).

For several decades, numerous studies have been devoted to understanding the mechanisms underlying opioids-induced hyperalgesia (OIH), including the central glutaminergic system (Mao, 2002; Mao et al., 2002; Gupta et al., 2011), spinal dynorphins (Gardell et al., 2002), descending facilitation (Barbaro et al., 1986; Heinricher et al., 1992; Morgan et al., 1992), decreased reuptake and enhanced nociceptive response, and genetic influences (Mao et al., 2002; King et al., 2005; Liang et al., 2006). Central sensitization, a common characteristic of OIH, is believed to reduce pain thresholds with the enhancement of excitatory neurotransmitters such as glutamate (Latremoliere and Woolf, 2009). Chronic morphine therapy leads to the downregulation of spinal glutamate transporters, which reduces the neuron’s ability to maintain *in vivo* glutamate homeostasis and enhances its hyperalgesia response to exogenous glutamate (Mao et al., 2002). Furthermore, *N*-methyl-D-aspartate (NMDA) receptor, which mediates the nociceptive effect of glutamate and plays pivotal roles in the development of hyperalgesia, may be activated by opioids. Conversely, the inhibition of NMDA receptor could prevent the development of OIH (Trujillo and Akil, 1991; Mao et al., 1994; Leal et al., 2010). NMDA receptor is composed of two NR1 and two NR2 subunits, and its functions depend on phosphorylation of these subunits (Fei and Tsien, 2009). The Ser890 and Ser896 of NR1 subunit can be phosphorylated by protein kinase C (PKC) and the Ser897 can be phosphorylated by protein kinase A (PKA) during the development of pain (Tingley et al., 1997; Zou et al., 2002). Furthermore, the NR2 subunit can be phosphorylated by Ca2+/calmodulin (CaM)−dependent protein kinase II (CaMKII) and PKC (MacDonald et al., 2001; Robison et al., 2005).

However, the common upstream mechanism underlying the activation of NMDA receptor after morphine administration remains unclear. A recent report showed that endoplasmic reticulum (ER) stress in the spinal cord contributed to the development of morphine tolerance (Liu et al., 2018). ER stress, which occurs due to the disturbances of ER homeostasis through means such as glucose deprivation, accumulation of unfolded or misfolded proteins, and oxidative stress (Xu et al., 2005; Bettigole and Glimcher, 2015), causes Ca^2+^ outflow and results in calcium overload in cells. CaMKII will be activated and bind to Ca^2+^/ CaM under the case of calcium loading (Meldolesi and Pozzan, 1998). Evidence also indicated that ER stress activated cAMP/PKA pathway and resulted in hepatic lipolysis (Deng et al., 2012; Song et al., 2017). Therefore, ER stress may play important roles in the phosphorylation of NMDA receptor and development of OIH. In this study, we want to answer why morphine treatment induces ER stress in neurons, how ER stress results in central sensitization, and how to attenuate OIH via new targets involved in ER stress signaling.

## Materials and Methods

### Ethics Approval and Consent to Participate

All procedures were strictly performed in accordance with the regulations of the ethics committee of the International Association for the Study of Pain and the Guide for the Care and Use of Laboratory Animals (The Ministry of Science and Technology of China, 2006). All animal experiments were approved by the Nanjing Medical University Animal Care and Use Committee and designed to minimize suffering and the number of animals used.

### Animals

Adult male CD-1 mice (18–22 g) and adult male Sprague-Dawley rats (200–250 g) were provided by the Experimental Animal Center at Nanjing Medical University, Nanjing, China. Animals were housed five to six per cage under pathogen-free conditions with soft bedding under a controlled temperature (22 ± 2°C) and a 12-hour light/dark cycle (lights on at 8:00 am). Behavioral testing was performed during the light cycle (between 9:00 am and 5:00 pm). The animals were allowed to acclimate to these conditions for at least 2 days before starting experiments. For each group of experiments, the animals were matched by age and body weight.

### Chemicals and Reagents

Morphine hydrochloride was purchased from Shenyang First Pharmaceutical Factory, Northeast Pharmaceutical Group Company (Shenyang, China). Glibenclamide, tauroursodeoxycholate, and salubrinal were purchased from MedChemExpress (Princeton, NJ, United States). Gliquidone, AEBSF, 4μ8c, and SCH77298 were purchased from Selleckchem (Houston, TX, United States). Antibody for β-actin was from Sigma-Aldrich (St. Louis, MO, United States). Antibodies for heat shock protein 70 (HSP70), Caspase-12, ATF6, p-eIF2α (Ser51), eIF2α, IRE1α, XBP1s, PKA, p-PKA (Thr197), ERK, p-ERK (Thr202/Tyr204), and NMDA Receptor subunit 1 (NR1) were from Cell Signaling Technology (Beverly, MA, United States). GRP78 and transferrin were from Abcam (Cambridge, MA, United States). Antibody for Phospho-NMDA Receptor subunit 1 (p-NR1) (Ser897) was purchased from Millipore (Billerica, MA, United States). Secondary antibodies for western blot were from Sigma-Aldrich (St. Louis, MO, United States). Immunofluorescent antibody for ionized calcium-binding adapter molecule1 (Iba-1) was from Abcam (Cambridge, MA, United States). Antibodies for glial fibrillary acidic protein (GFAP) and neuronal nuclear protein (NeuN) were from Millipore (Billerica, MA, United States). Secondary antibodies for immunofluorescence were from Jackson Immunoresearch Laboratories (West Grove, PA, United States) and Abcam (Cambridge, MA, United States). Fetal bovine serum (FBS), cell culture media and supplements were purchased from Gibco.

### Opioids-Induced Hyperalgesia Model and Behavioral Analysis

Animals were habituated in the testing environments for 2 days and carried out behavioral testing in a blinded manner. Mice and rats were injected with different doses of morphine. Mice were injected subcutaneously with morphine (5 mg/kg) twice daily for 6 days to induce OIH. Behavioral testing was performed before the first morphine administration every morning by tail-flick assay. The tail-flick test was performed using a water bath with temperature maintained at 48°C. Each animal was gently wrapped in a cloth by the experimenter. The distal one-third of tail was immersed in a water bath set at 48°C, and mice rapidly removed their tail from the bath at the first sign of the discomfort. The chronometer was stopped as soon as the mouse withdrew its tail from the hot water and the latency time was recorded (in seconds). A cut-off time of 20 s was set to avoid tissue damage. Different doses of glibenclamide (0.08, 0.4 or 2 μg/10 μL) were administered by intrathecal injection 15 min before morphine administration. AEBSF (10 μg/10 μL), 4μ8c (2 μg/10 μL), tauroursodeoxycholate(100 μg/10 μL) and Salubrinal (2 μg/10 μL) were administered by intrathecal injection 15 min before morphine administration. Rats were subcutaneously injected with morphine (7.5 mg/kg) twice daily for six consecutive days.

### Cell Cultures

SH-SY5Y cells were maintained in humidified 5% CO_2_ at 37°C in Modified Eagle Media: F-12 (MEM/F12, Gbico, NY, United States) supplemented with 10% (v/v) FBS (Gibco), 80 U/mL penicillin, and 0.08 mg/mL streptomycin. For further experiments, SH-SY5Y cells were plated in 6-well plate overnight and then treated with morphine (200 μM) the following morning with or without glibenclamide (200 μM), gliquidone (200 μM), AEBSF (200 μM), tauroursodeoxycholate (500 μM), 4μ8c (10 μM), and SCH772984 (2 μM) for 12 h. Cell extracts and precipitated supernatants were analyzed by immunoblot assay.

### Intrathecal Injection Procedure

To perform intrathecal (*i.t*.) injections, the mice were placed in a prone position and the midpoint between the tips of the iliac crest was located. A Hamilton syringe with a 30-gauge needle was inserted into the subarachnoid space of the spinal cord between the L5 and L6 spinous processes. Proper intrathecal injection was systemically confirmed by observation of a tail flick. Intrathecal injection did not affect baseline responses, compared with latencies recorded before injection.

### Western Blot

Samples (cells or spinal cord tissue segments at L1-L6) were collected and washed with ice-cold PBS before being lysed in radio immunoprecipitation assay (RIPA) lysis buffer and then sample lysates were separated by SDS-PAGE and electrophoretically transferred onto polyvinylidene fluoride membranes (Millipore). The membranes were blocked with 10 % low-fat dry powdered milk or with 5% BSA and 5% low-fat dry powdered milk in TBST (Tris–HCl, NaCl, Tween 20) for 2 h at room temperature, and then probed with primary antibodies at 4°C overnight. Finally, the horseradish peroxidase (HRP)-coupled secondary antibodies were utilized for detecting corresponding primary antibody. The primary antibodies utilized included β-actin (1:5000), Caspase-12 (1:1000), HSP70 (1:1000), GRP78 (1:500), ATF6 (1:1000), p-eIF2α (Ser51) (1:1000), eIF2α (1:1000), IRE1α (1:1000), XBP1s (1:1000), ERK (1:1000), p-ERK (Thr202/Tyr204) (1:1000), PKA (1:1000), p-PKA(Thr197) (1:1000), NMDA Receptor subunit 1 (NR1) (1:1000), and Phospho-NMDA Receptor subunit 1 (p-NR1) (Ser897) (1:1000). The bands were then developed by enhanced chemiluminescence reagents (PerkinElmer, Waltham, MA, United States). Data were analyzed with the Molecular Imager and the associated software Image J (NIH, United States). The original unprocessed images of all western blots were provided in Supplementary Data Sheets S1–S10.

### Immunohistochemistry

Under deep anesthesia by intraperitoneal injection of pentobarbital sodium (50 mg/kg), animals were perfused with normal saline followed by 4 % paraformaldehyde in 0.1 M PBS, pH 7.2–7.4, for 20 min. Then L4 and L5 lumbar segments were dissected out and post-fixed in the same fixative. The embedded blocks were sectioned as 25 μm thick and processed for immunofluorescence assay. Sections from each group (four mice in each group) were incubated with primary antibodies: GRP78 (1:200), Iba-1 (1:200), NeuN (1:200), and GFAP (1:200). Then the free-floating sections were washed with PBS and incubated with the secondary antibodies (1:300) for 2 h at room temperature. After being washed three times with PBS, the samples were investigated with a confocal microscopy (Zeiss LSM710, Germany).

### Overexpression of Adenovirus-Mediated HSP70 in the Spinal Cord

The adenovirus-HSP70 or adenovirus vector were purchased from OBiO Technology (Shanghai) Corp., Ltd. Delivery of adenovirus into the spinal cord was conducted by intrathecal injection for three times, at one day before the first morphine administration and at 1 and 3 days after the first morphine administration. 10 μL of adenovirus was intrathecally injected into the lumbar spinal cord of mice each time.

### Collection of Cerebrospinal Fluid (CSF)

Adult male Sprague-Dawley rats (200–250 g) were housed under a 12 light/dark cycle, with food and water available ad lib. The animals were anesthetized with pentobarbital sodium (50 mg/kg, *i.p*.). The CSF was carefully collected from the cisterna magna of each rat, as described previously (Hudson et al., 1994), and inspected for blood contamination. Contaminated samples were discarded. Approximately 80 μL of CSF was collected from each animal. After a short centrifugation step (5 min at 5000 × *g*, 4°C), the samples were dissolved in 2 × SDS loading buffer, boiled, and analyzed by SDS-PAGE followed by western blotting.

### Statistical Analysis

GraphPad Prism 7 software (GraphPad Software, San Diego, CA, United States) was used to conduct all the statistical analyses. The differences between two groups were evaluated by Student’s *t*-test. The data from more than two groups were evaluated by One-Way ANOVA. Results were represented as mean ± SEM of the independent experiments. Results described as significant were based on a criterion of *P* < 0.05.

## Results

### Chronic Morphine Treatment Induces Hyperalgesia and Evokes ER Stress in Neurons of Spinal Cord in Mice

**FIGURE 1:**
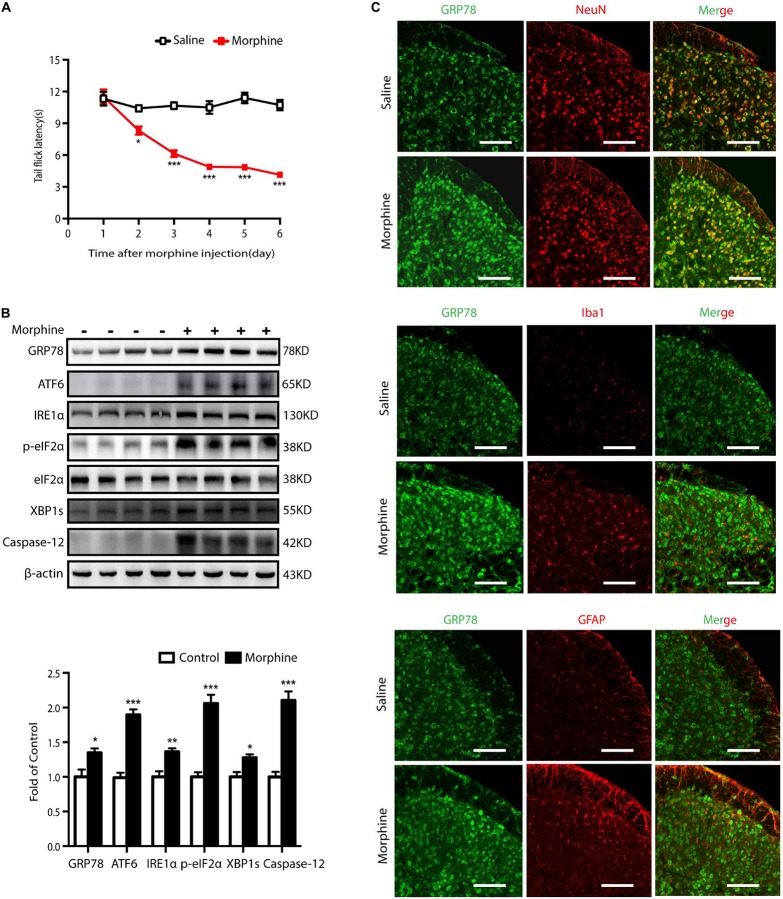
Chronic morphine treatment induces hyperalgesia and evokes ER stress in neurons of spinal cord in mice. **(A)** Chronic morphine treatment induced hyperalgesia in mice. Mice were subcutaneously injected with saline or morphine (5 mg/kg) twice daily for 6 consecutive days. Behavioral testing was conducted before first morphine administration every morning by tail-flick assay. Chronic administration of morphine significantly decreased tail flick latency from days 1 to 5. The saline-treated group served as control. Data was analyzed by Student’s *t* test (*n* = 8) (*P < 0.01, ***P < 0.001 vs. control). **(B)** Representative western blot image and analysis to quantify ER stress-related molecule levels in the spinal cord. Consecutive administration of morphine (5 mg/kg) twice daily for 6 days evokes ER stress in the spinal cord. The expression of GRP78, ATF6, IRE1α, p-eIF2, XBP1s, Caspase-12 were significantly increased in OIH mice measured by western blots (*n* = 4). The spinal samples were collected 1 h after the last morphine treated and determined by western blot. Significant difference was revealed following Student’s *t* test (*P < 0.05, **P < 0.01, ***P < 0.001 vs. control). **(C)** Distribution and cellular localization of GRP78 in the dorsal horn of the spinal cord before or after morphine administration. The level of GRP78 (green) was significantly increased in OIH mice compared with those in control mice. Double immunostaining of GRP78 and specific makers in OIH mice showed that GRP78 was co-localized with NeuN (red) not with astrocytic maker GFAP (red) or microglial maker Iba1 (red). Spinal samples were collected after the last administration of morphine. Scale bar: 100 μm.

Firstly, the animal model for OIH was utilized to study the effects of morphine on ER stress. Mice were subcutaneously injected with saline or morphine (5 mg/kg) twice daily for six consecutive days. Behavioral testing was conducted before morphine administration every morning by tail-flick assay. As shown in Figure 1A, the tail flick latencies in mice receiving morphine gradually decreased and were significantly lower than those in saline mice from day one to day 5 (*p* < 0.05). These results indicated that morphine induced hyperalgesia in mice. To study the involvement of ER stress in OIH mice, we examined the spinal expression of GRP78, a marker of ER stress (Bertolotti et al., 2000). On day 5, the level of GRP78 was increased in the spinal cords from mice treated with morphine, implying that chronic morphine treatment might trigger ER stress (Figure 1B). When ER stress is induced, it can cause unfolded protein response (UPR) through three major transducers: activating transcription factor 6 (ATF6), inositol-requiring ER-to-nucleus signal kinase 1 (IRE1α), and RNA-dependent protein kinase-like ER kinase (PERK) (Endo et al., 2007). Here, immunoblot results indicated that the levels of ATF6, IRE1α, p-eIF2, XBP1s, and Caspase-12 were increased in the spinal cord of mice under the treatment of morphine (Figure 1B).

Then, we tested the cellular localization and distribution of GRP78 in the dorsal horn of the spinal cord. The immunoreactivity of GRP78 in OIH mice was higher than those in saline-treated mice. Furthermore, cellular distribution of GRP78 measured by confocal microscopic scanning showed that GRP78 mainly co-localized with neuronal marker NeuN, but not with microglial marker Iba1 or astrocytic marker GFAP (Figure 1C). These results indicate a significant upregulation of ER stress in neurons in the development of OIH.

### ER Stress in OIH Mice Was Mediated by ATF6 and IRE1α

**FIGURE 2:**
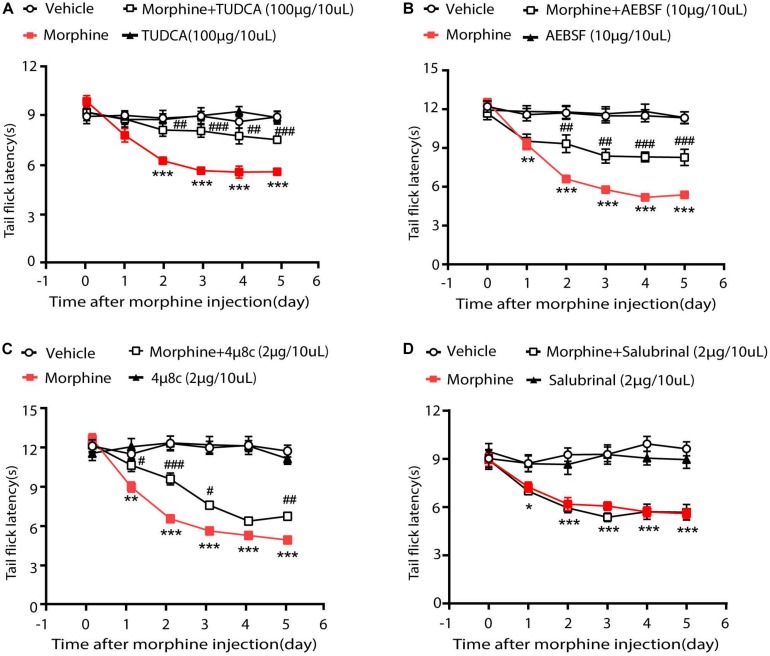
ER stress in OIH mice is mediated by ATF6 and IRE1α. The behavior testing was performed to evaluate the effects of UPR-related three pathways on OIH. **(A)** Pretreatment with TUDCA could attenuate the development of OIH in mice. Mice were subcutaneously injected with saline or morphine (5 mg/kg) twice daily for 6 consecutive days. TUDCA (100 μg/10 μL) was administered by intrathecal injection 30 min before morphine administration. **(B)** Pretreatment with AEBSF could attenuate the development of OIH in mice. Mice were subcutaneously injected with saline or morphine (5 mg/kg) twice daily for 6 consecutive days. AEBSF (10 μg/10 μL) was administered by intrathecal injection 30 min before morphine administration. **(C)** Pretreatment with 4μ8C could attenuate the development of OIH in mice. Mice were subcutaneously injected with saline or morphine (5 mg/kg) twice daily for 6 consecutive days. 4μ8C (2 μg/10 μL) was administered by intrathecal injection 30 min before morphine administration. **(D)** Pretreatment with Salubrinal could not attenuate the development of OIH in mice. Mice were subcutaneously injected with saline or morphine (5 mg/kg) twice daily for 6 consecutive days. Salubrinal (2 μg/10 μL) was administered by intrathecal injection 30 min before morphine administration. D data were analyzed by one-way ANOVA (*P < 0.05, **P < 0.01, ***P < 0.001 vs. vehicle, ^#^*P* < 0.05, ^##^*P* < 0.01, ^###^*P* < 0.001 vs. morphine-treated group). Behavioral testing was performed before first morphine administration every morning by tail-flick assay (*n* = 8).

Then we investigated whether ER stress-related signaling pathways contributed to the development of OIH. Therefore, TUDCA, a chemical suppressor of ER stress (Kawasaki et al., 2012; Rani et al., 2017), was given via intrathecal injection 30 min before the administration of morphine. As is shown in Figure 2A, consecutive treatments with TUDCA (100 μg/10 μL) could significantly increase the tail flick latency by 58.25% in mice treated with morphine. To examine the roles of ATF6, IRE1α, and PERK during OIH, their specific inhibitors were, respectively, administrated (*i.t.*) to mice 30 min before morphine administration. It was shown that AEBSF (10 μg/10 μL, targeting ATF6) and 4μ8C (2 μg/10 μL, targeting IRE1α) increased the tail flick latency by 26.6 and 48.5%, respectively, in morphine-treated mice (Figures 2B,C). However, salubrinal (2 μg/10 μL, the specific inhibitor on PERK-eIF2α signaling) did not increase the tail flick latency in morphine-treated mice (Figure 2D). Taken together, these results indicated that OIH resulted from ER stress may be mediated via ATF6 and IRE1α pathway but not via PERK pathway.

### Morphine-Induced ER Stress Enhances PKA-Mediated Phosphorylation of NMDA Receptor

**FIGURE 3:**
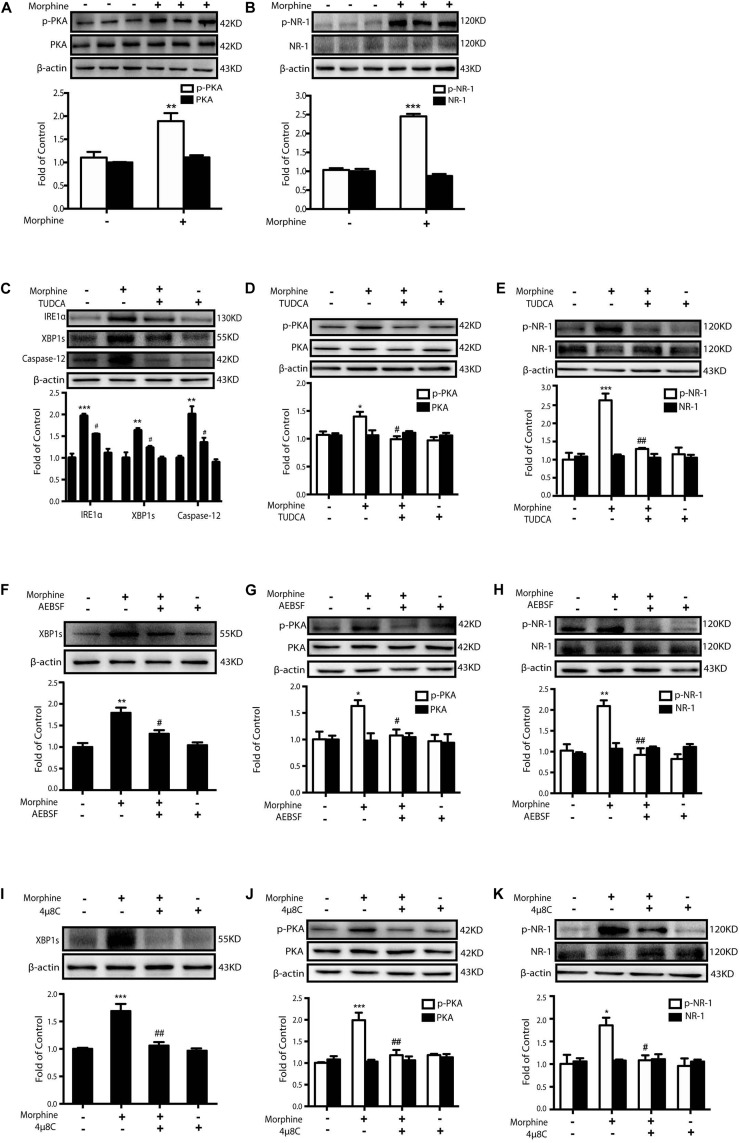
Morphine-induced ER stress enhances PKA-mediated phosphorylation of NMDA receptor. Representative western blot images were shown in this figure. **(A,B)** Morphine induced the phosphorylation of PKA and NR-1 in SH-SY5Y cells. Cells were collected 12 h after morphine (200 μM) treatment and analyzed by western blot (*n* = 3). **(A,B)** data were analyzed by Student’s *t* test (**P < 0.01, ***P < 0.001 vs. control). **(C–E)** Pretreatment with TUDCA could inhibit the up-regulation of ER stress-related molecule IRE1α, XBP1s and Caspase-12 induced by morphine in SH-SY5Y cells. TUDCA also suppressed the phosphorylation of PKA and NR-1 induced by morphine in SH-SY5Y cells. TUDCA (500 μM) was given 15 min before morphine (200 μM) administration. Cells were collected 12h after morphine treatment and analyzed by western blot (*n* = 3). **(F–H)** AEBSF inhibited the up-regulation of XBP1s induced by morphine in SH-SY5Y cells. AEBSF also suppressed the phosphorylation of PKA and NR-1induced by morphine in SH-SY5Y cells. AEBSF (200 μM) was given 15 min before morphine (200 μM) administration. Cells were collected 12 h after morphine treatment and analyzed by western blot (*n* = 3). **(I–K)** 4μ8C decreased the level of XBP1s induced by morphine in SH-SY5Y cells. 4μ8C also inhibited the phosphorylation of PKA and NR-1 induced by morphine in SH-SY5Y cells. 4μ8C (IRE1α inhibitor, 10 μM) was given 15 min before morphine (200 μM, 12 h) administration. Cells were collected 12 h after morphine treatment and analyzed by western blot (*n* = 3). **(C–K)** data were analyzed by one-way ANOVA (*P < 0.05, **P < 0.01, ***P < 0.001 vs. control, ^#^*P* < 0.05, ^##^*P* < 0.01 vs. morphine-treated group).

It was reported that PKA caused the phosphorylation of NR-1 subunit Ser897 site of NMDA receptor upon the stimulation of glutamate (Mao et al., 2009) in pain development. Then we questioned whether ER stress induced by morphine treatment could cause PKA-mediated activation of NMDA receptor. The SH-SY5Y neuronal cell line which endogenously expressed opioids receptors (Wang and Traynor, 2013) was utilized to investigate whether morphine could enhance PKA-mediated phosphorylation of NMDA receptor. After incubation with morphine (200 μM) for 12 h, SH-SY5Y cells showed an increased phosphorylation of PKA and NR-1 Ser897 (Figures 3A,B). Then the ER-stress inhibitor TUDCA, a serine protease inhibitor AEBSF (inhibiting the transcriptional induction of ATF6-target genes) (Okada et al., 2003) or IRE1α inhibitor 4μ8C was administrated 15 min prior to morphine (200 μM, 12 h), respectively. Pretreatment with TUDCA (500 μM) could inhibit the up-regulation of IRE-1α, XBP1s, and Caspase-12 in SH-SY5Y cells stimulated with morphine (Figure 3C). Phosphorylation of PKA and NR-1 was also inhibited by TUDCA (Figures 3D,E). Similarly, AEBSF (200 μM) and 4μ8C (10 μM)could not only downregulate the level of XBP1s, but also inhibit the phosphorylation of PKA and NR-1 caused by morphine (Figures 3F–K). These results demonstrate that it is morphine-induced ER stress which enhances phosphorylation of PKA and activation of NMDA receptors.

### Morphine-Induced Release of HSP70 Is Required for ER Stress

**FIGURE 4:**
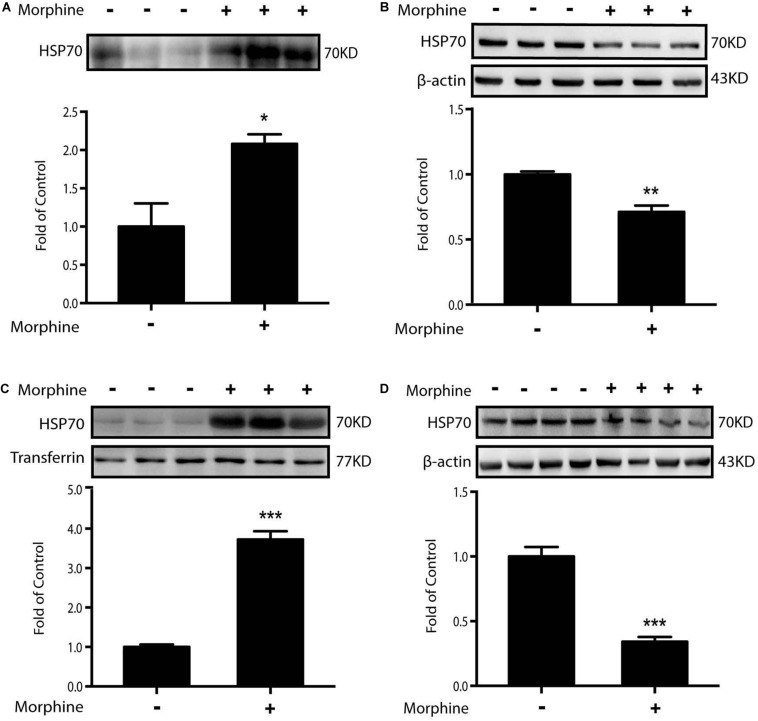
Morphine induces the release of HSP70 *in vivo* and *in vitro.* Representative western blot images were shown in this figure. **(A)** Morphine induced the efflux of HSP70 into extracellular environment in SH-SY5Y cells. Supernatants were collected 12 h after morphine (200 μM) administration and measured by western blot (*n* = 3). **(B)** Morphine decreased the intracellular protein level of HSP70 in SH-SY5Y cells. Cell extracts were collected 12 h after morphine (200 μM) treatment and analyzed by western blot (*n* = 3). **(C)** Rats were subcutaneously injected with morphine (7.5 mg/kg) twice daily for 6 consecutive days. Consecutive administration of morphine induced the release of HSP70 in CSF. CSF was collected from rats 1 h after the last administration and determined by western blot (*n* = 6). Transferrin is used as a loading control. **(D)** Consecutive administration of morphine (5 mg/kg) twice daily for 7 days in mice induced the release of HSP70 in the spinal cord. The spinal samples were collected 1 h after the last morphine treated and determined by western blot (*n* = 4). **(A–D)** data were analyzed by Student’s *t* test (*P < 0.05, **P < 0.01, ***P < 0.001 vs. control).

According to the above data, we raised a question as to why morphine could induce ER stress. HSP70, an abundant and quickly inducible protein, is constitutively expressed at normal growth temperatures and functions as a molecular chaperone supporting the folding and transport of newly synthesized polypeptides (Hartl and Martin, 1996; Hartl and Hayer-Hartl, 2002; Bertelsen et al., 2009; Venetianer et al., 2013). Moreover, in our previous study, we found that chronic morphine administration induced the release of HSP70 from spinal neurons (Qu et al., 2017). Weighing on the role of HSP70, we incubated SH-SY5Y cells with morphine for 12 h, then the supernatants were collected and analyzed by western blot. It was showed that morphine induced the efflux of HSP70 into supernatant (Figure 4A). Simultaneously, morphine lowered the protein level of HSP70 in SH-SY5Y cells (Figure 4B). Then, we collected the cerebrospinal fluid (CSF) from OIH rats. Immunoblot data showed that HSP70 was significantly released into CSF, with a notable decrease in spinal cords after morphine treatment (Figures 4C,D). On the basis of these results, whether the release of HSP70 was required for ER stress would be tested.

**FIGURE 5:**
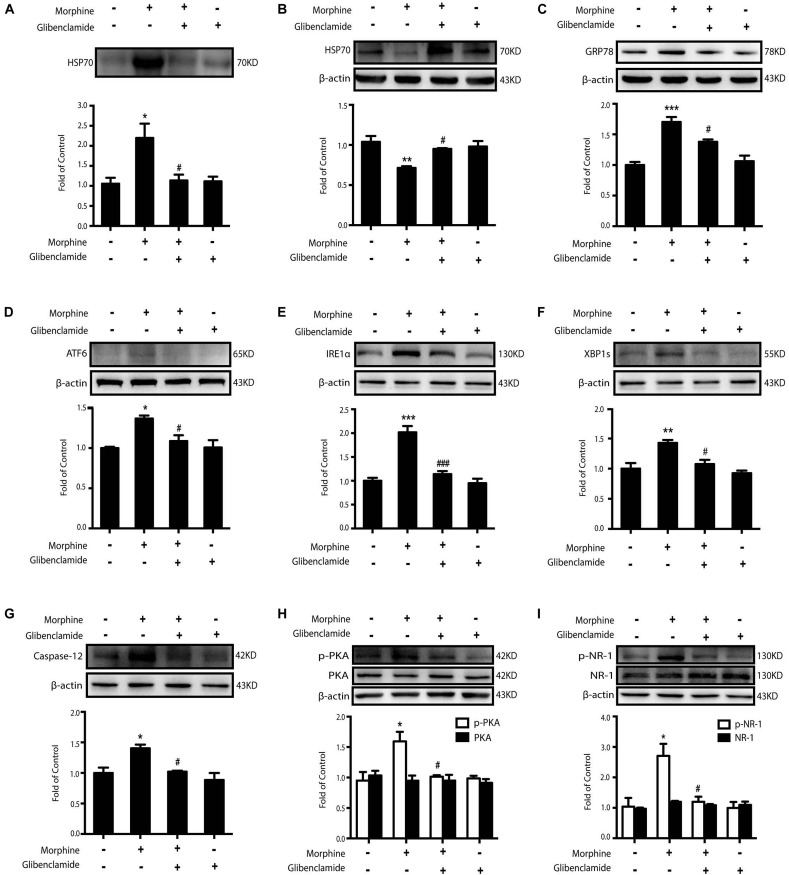
The inhibition of HSP70 releasing by glibenclamide suppresses morphine-induced ER stress and the phosphorylation of PKA and NR-1. Representative western blot images were shown in this figure. **(A)** Glibenclamide administration (200 μM) 15 min prior to morphine (200 μM, 12 h) prevented the morphine-induced HSP70 release in SH-SY5Y cells. Supernatants were collected 12 h after morphine treatment and determined by western blot (*n* = 3). **(B)** Glibenclamide (200 μM) inhibited the decrease of intracellular HSP70 caused by morphine in SH-SY5Y cells. Cell extracts were collected 12 h after morphine treatment and analyzed by western blot (*n* = 3). **(C–G)** Pretreatment with gibenclamide abrogated the up-regulation of ER stress molecule GRP78, ATF6, IRE1α, XBP1s and Caspase-12 induced by morphine in SH-SY5Y cells. Glibenclamide (200 μM) was given 15 min before morphine (200 μM, 12 h) administration. Cells were collected and analyzed by western blot (*n* = 3). **(H)** Glibenclamide abolished the phosphorylation of PKA induced by morphine treatment in SH-SY5Y cells. Glibenclamide (200 μM) was given 15 min before morphine (200 μM, 12 h) administration. Cells were collected 12 h after morphine treatment and analyzed by western blot (*n* = 3). **(I)** Glibenclamide could inhibit the phosphorylation of NR-1 induced by morphine treatment in SH-SY5Y cells. Glibenclamide (200 μM) was given 15 min before morphine (200 μM, 12 h) administration. Cells were collected and analyzed by western blot (*n* = 3). A-I data were analyzed by one-way ANOVA (*P < 0.05, **P < 0.01, ***P < 0.001 vs. control, ^#^*P* < 0.05, ^###^*P* < 0.001 vs. morphine-treated group).

In our previous study, we found that HSP70 could be released via the ATP-binding cassette (ABC) transporter proteins. Morphine could activate K_ATP_ channel, which is an ABC transporter protein (Mambula and Calderwood, 2006; Qu et al., 2017). Therefore, glibenclamide, a classic K_ATP_ channel blocker, was utilized to inhibit the release of HSP70 caused by morphine. We found that the level of extracellular HSP70 was decreased (Figure 5A) and glibenclamide (200 μM) reversed the decline of intracellular HSP70 upon morphine stimulation in SH-SY5Y cells (Figure 5B). Further, pretreatment with glibenclamide could inhibit the up-regulation of ER stress-related molecules GRP78, ATF6, IRE1α, XBP1s, and Caspase-12 and suppress the phosphorylation of PKA and NR-1 in SH-SY5Y cells treated with morphine (Figures 5C–I). In order to further verify that glibenclamide alleviated ER stress via blocking K_ATP_ channel, we used another sulfonylurea K_ATP_ channel blocker gliquidone. Data showed that gliquidone (200 μM) prevented the release of HSP70 caused by morphine (200 μM, 12 h) in SH-SY5Y cells, too (Supplementary Figure S1). As glibenclamide, gliquidone showed the same effects on ER stress and NR1 phosphorylation in morphine-treated SH-SY5Y cells.

**FIGURE 6:**
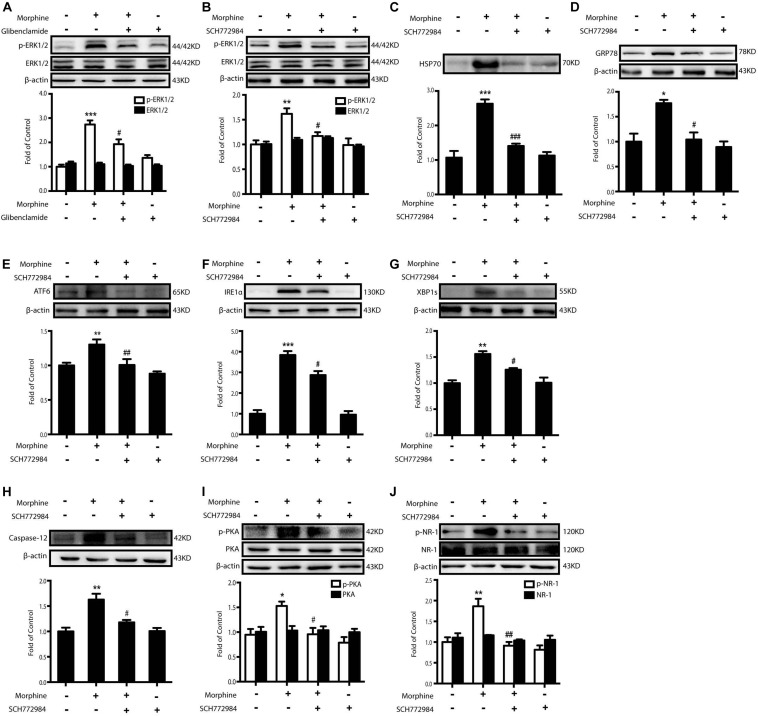
The inhibition of ERK1/2 suppresses morphine-induced releasing of HSP70 and attenuates the ER stress and the phosphorylation of PKA and NR-1. Representative western blot images were shown in this figure. **(A)** Glibenclamide abolished the phosphorylation of ERK1/2 elevated by morphine. Glibenclamide (200 μM) was given 15 min before morphine (200 μM, 1 h) administration. Cell extracts were collected and analyzed by western blot (*n* = 3). **(B)** ERK1/2 inhibitor, SCH772984, could suppress the phosphorylation of ERK1/2 induced by morphine treatment in SH-SY5Y cells. SCH772984 (2 μM) was given 15 min before morphine (200 μM, 1 h) administration. Cells were collected 12 h after morphine treatment and analyzed by western blot (*n* = 3). **(C)** HSP70 release induced by morphine was reversed by ERK1/2 inhibitor in SH-SY5Y cells. SCH772984 (2 μM) was given 15 min before morphine (200 μM, 12 h) administration. Supernatants were collected and analyzed by western blot (*n* = 3). **(D–H)** Pretreatment with ERK1/2 inhibitor could inhibit the up-regulation of ER stress molecule GRP78, ATF6, IRE-1α, XBP1s and Caspase-12 induced by morphine in SH-SY5Y cells. SCH772984 (2 μM) was given 15 min before morphine (200 μM, 12 h) administration. Cells were collected 12 h after morphine treatment and analyzed by western blot (*n* = 3). **(I)** ERK1/2 inhibitor decreased the phosphorylation of PKA induced by morphine in SH-SY5Y cells. SCH772984 (2 μM) was given 15 min before morphine (200 μM, 12 h) administration. Cells were collected 12 h after morphine treatment and analyzed by western blot (*n* = 3). **(J)** Pretreatment with ERK1/2 inhibitor could inhibit the phosphorylation of NR-1 induced by morphine in SH-SY5Y cells. SCH772984 (2 μM) was given 15 min before morphine (200 μM, 12 h) administration. Cells were collected 12 h after morphine treatment and analyzed by western blot (*n* = 3). **(A–J)** data were analyzed by one-way ANOVA (*P < 0.05, **P < 0.01, ***P < 0.001 vs. control, ^#^*P* < 0.05, ^##^*P* < 0.01, ^###^*P* < 0.01 vs. morphine-treated group).

The next question was how morphine induced the release of HSP70 by K_ATP_ channel. Previous studies demonstrated that ERK1/2 mediated stress-induced release of HSP70 and the phosphorylation level of ERK1/2 was markedly elevated in SH-SY5Y cells 60 min after morphine (200 μM) administration (Taylor et al., 2010; Qu et al., 2017). We found that the level of ERK1/2 phosphorylation induced by morphine was inhibited by glibenclamide (200 μM) pretreatment (15 min) in SH-SY5Y cells (Figure 6A). These data indicated that the activation of ERK1/2 might be downstream to K_ATP_ channel. Therefore, we wanted to investigate whether or not the inhibition of ERK1/2 could suppress the excretion of HSP70. ERK1/2 inhibitor, SCH772984 (2 μM) was given 15 min before morphine administration. It decreased the phosphorylation of ERK1/2 (Figure 6B) and suppressed the efflux of HSP70 (Figure 6C). Moreover, pretreatment with ERK1/2 inhibitor could inhibit the ER stress-related molecules increased by morphine in SH-SY5Y cells (Figures 6D–H). SCH772984 also inhibited the phosphorylated PKA and NR-1 stimulated by morphine in SH-SY5Y cells (Figures 6I,J).

**FIGURE 7:**
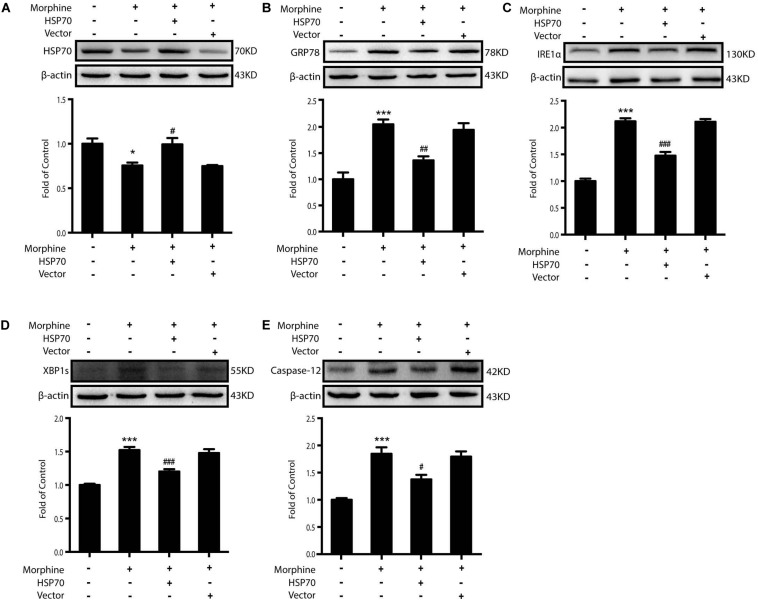
Overexpression of HSP70 can improve ER stress-induced by morphine in SH-SY5Y cells. Representative western blot images were shown in this figure. **(A)** Adenovirus-mediated HSP70 overexpression inhibited the decrease of intracellular HSP70 caused by morphine in SH-SY5Y cells. Cell extracts were collected 12 h after morphine treatment and analyzed by immunoblot assay (*n* = 3). **(B–E)** Adenovirus-mediated HSP70 rescue could inhibit the up-regulation of ER stress molecule GRP78, IRE1α, XBP1s and Caspase-12 induced by morphine in SH-SY5Y cells. Cells were collected 12 h after morphine treatment and analyzed by western blot (*n* = 3). **(A–E)** data were analyzed by one-way ANOVA (*P < 0.05, ***P < 0.001 vs. control, ^#^*P* < 0.05, ^##^*P* < 0.01, ^###^*P* < 0.001 vs. morphine-treated group).

Then we sought to examine whether HSP70 overexpression could improve ER stress in SY-SY5Y cells. We utilized adenovirus-mediated HSP70 overexpression (Figure 7A) and found that improved intracellular HSP70 inhibited the up-regulation of IRE1α, XBP1s, and Caspase-12 induced by morphine in SH-SY5Y cells (Figures 7B–E). Taken together, these results revealed that morphine induced the release of HSP70 and is required for ER stress, and the rescue of HSP70 could improve ER stress in SH-SY5Y cells.

### The Rescue of HSP70 in the Spinal Cord Improves OIH in Rodents

**FIGURE 8:**
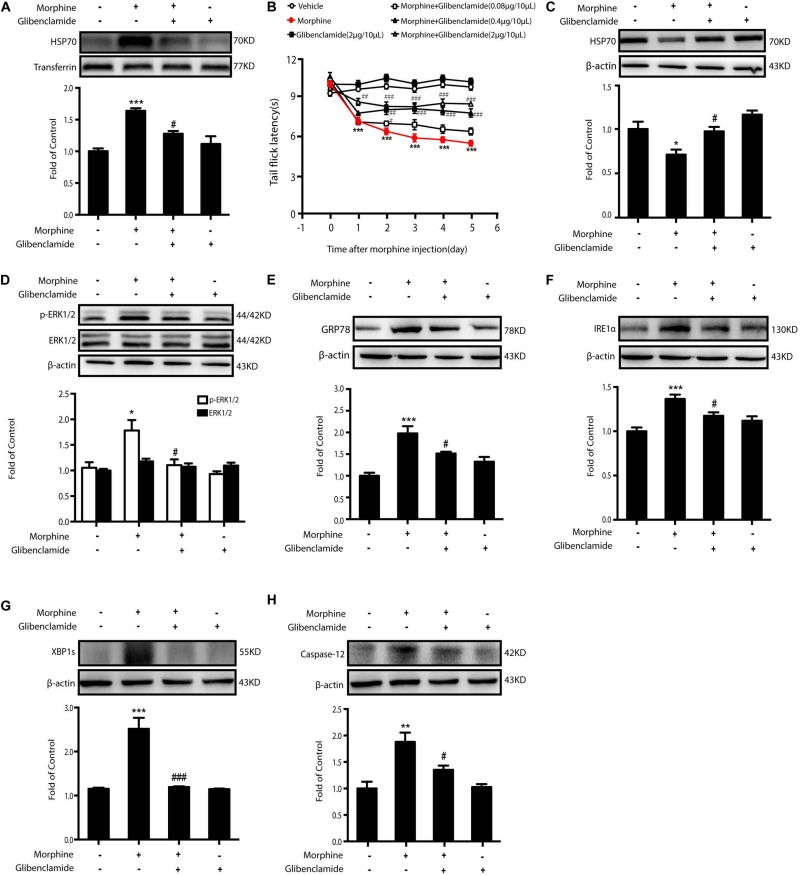
The inhibition of HSP70 releasing by glibenclamide in the spinal cord of animals improves OIH. Representative western blot images were shown in this figure. **(A)** Consecutive administration of glibenclamide (2 μg/10 μL, *i.t*.) for 6 days inhibited the release of HSP70 induced by morphine (10 μg/10 μL, *i.t*.) in CSF. CSF was collected from rats 1 h after the last administration and determined by western blot (*n* = 4). Transferrin was used as a loading control. Data were analyzed by one-way ANOVA (***P < 0.001 vs. control, ^#^*P* < 0.05 vs. morphine-treated group). **(B)** Pretreatment with glibenclamide could inhibit the development of OIH in mice. Mice were subcutaneously injected with saline or morphine (5 mg/kg) twice daily for 6 consecutive days. Different doses of glibenclamide (0.08, 0.4, and 2 μg/10 μL, *i.t*.) was administered by intrathecal injection 15 min before morphine administration. Before first morphine administration every morning, behavioral test was evaluated by tail-flick assay (*n* = 8). Data were analyzed by one-way ANOVA (*P < 0.05, **P < 0.01, ***P < 0.001 vs. vehicle, ^###^*P* < 0.001 vs. morphine-treated group). **(C)** Pretreatment of glibenclamide suppressed the decrease of HSP70 induced by morphine in the spinal cord (*n* = 4). Glibenclamide (0.08, 0.4, and 2 μg/10 μL, *i.t*.) was administered once daily for 6 days. One hour after the final administration, spinal samples were collected and determined by western blot. **(D)** Administration of glibenclamide inhibited the phosphorylation of ERK1/2 elevated by morphine in the spinal cord. Glibenclamide (0.08, 0.4, and 2 μg/10 μL, *i.t*.) was administered once daily for 6 days. One hour after the final administration, spinal samples were collected and determined by western blot. **(E–H)** Administration of glibenclamide inhibited the increase of GRP78, IRE1α, XBP1s and Caspase-12 induced by morphine in the spinal cord. Glibenclamide (0.08, 0.4, and 2 μg/10 μL, *i.t*.) was administered once daily for 6 days. One hour after the final administration, spinal samples were collected and determined by western blot. C-H data were analyzed by one-way ANOVA (*P < 0.05, **P < 0.01, ***P < 0.001 vs. control, ^#^*P* < 0.05, ^###^*P* < 0.001 vs. morphine-treated group).

As morphine-induced ER stress was alleviated by K_ATP_ block or HSP70 overexpression *in vitro*, we further investigated the effects of HSP70 rescue *in vivo.* Glibenclamide was given to OIH animals first. Consecutive administration of glibenclamide (2 μg/10 μL, *i.t.*) for 6 days inhibited the release of HSP70 in CSF (Figure 8A) and improved the analgesic behaviors in OIH mice administered with morphine in a dose-dependent manner (Figure 8B), although there was no significant difference in mice treated with glibenclamide alone. Besides, consecutive administration of glibenclamide abolished the reduction of HSP70 (Figure 8C), downregulated the phosphorylation of ERK1/2 (Figure 8D), and inhibited the increased expression of GRP78, IRE1α, XBP1s, and Caspase-12 (Figures 8E–H) in the spinal cord from mice with chronic morphine treatment.

**FIGURE 9:**
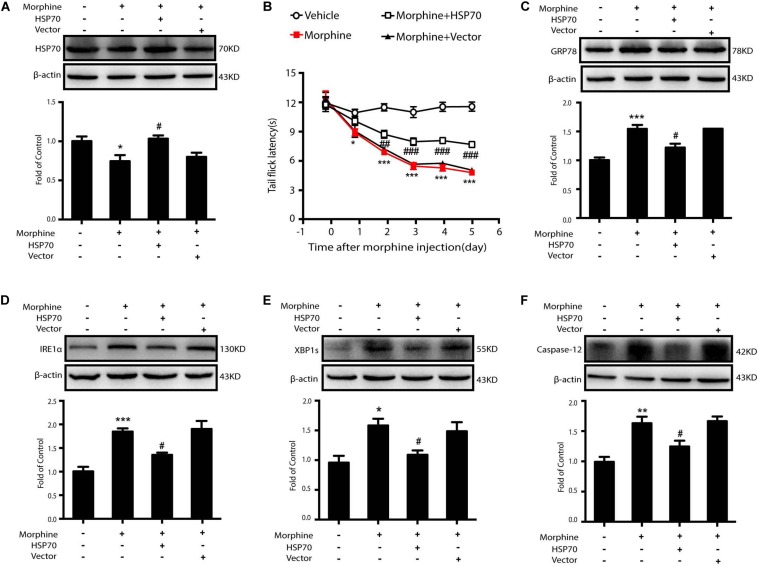
The rescue of HSP70 in the spinal cord of mice improves OIH. Representative western blot images were shown in this figure. **(A)** Adenovirus-mediated HSP70 rescue attenuated the decrease of HSP70 in the spinal cord induced by morphine (*n* = 4). Mice were subcutaneously injected morphine (5 mg/kg) twice daily for 6 consecutive days. Adenovirus-HSP70 was administered by intrathecal injection for three times, at 1 day before the first morphine administration and at 1 and 3 days after the first morphine administration. 10 μL of adenovirus was intrathecally injected into lumbar spinal cord of mice for every time. Data were analyzed by one-way ANOVA (*P < 0.05 vs. control, ^#^*P* < 0.05 vs. morphine-treated group). **(B)** The rescue of HSP70 in the spinal cord could inhibit the development of OIH in mice. Behavioral testing was conducted before first morphine administration every morning by tail-flick assay (*n* = 8). Data were analyzed by one-way ANOVA (*P < 0.05, ***P < 0.001 vs. vehicle, ^#^*P* < 0.05, ^##^*P* < 0.01, ^###^*P* < 0.001 vs. morphine-treated group). **(C–F)** The rescue of HSP70 in the spinal cord could suppress the increase of GRP78, IRE1α, XBP1s, Caspase-12 induced by morphine in the spinal cord (*n* = 4). One hour after the final administration, spinal samples were collected and determined by western blot. **(C–F)** data were analyzed by one-way ANOVA (*P < 0.05, **P < 0.01, ***P < 0.001 vs. control, ^#^*P* < 0.05 vs. morphine-treated group).

Then, 10 μL of adenovirus-HSP70 or adenovirus vector was intrathecally given to OIH mice for three times, 24 h before, 24 h after, and 72 h after the first morphine administration. The behavioral assessment demonstrated that overexpressed HSP70 could significantly suppress OIH in mice (Figures 9A,B). Furthermore, the levels of GRP78, IRE1α, XBP1s, and Caspase-12 were decreased in mice with adenovirus-mediated HSP70 overexpression (Figures 9C–F).

## Discussion

In the present study, the principal findings are: (1) during the development of OIH, ER is robustly induced by morphine in the neurons in the spinal cord, with activation of all the three UPR pathways, including IRE1α/XBP1s, ATF6, and PERK/eIF2α. However, only inhibitions on IRE1α/XBP1s or ATF6, but not on PERK/eIF2α, can block UPR cascades and consequently suppress the development of OIH; (2) Morphine induces the release of HSP70 to extracellular environments and decreases its cytosolic level via K_ATP_/ERK pathway. Lack of HSP70 directly induces UPR in ER; (3) ER stress in neurons later results in PKA phosphorylation and NR1 activation, which indicates central sensitization and occurrence of OIH in rodents.

The mechanism underlying OIH is complex and far beyond having a clear explanation. Here, we demonstrated a distinct pathway in which ER stress may be the essential node from morphine stimulation to central sensitization in neurons. As a standard indicator of ER stress and UPR initiation, GRP78 was up-regulated by morphine administration in the spinal neurons. TUDCA, a chemical chaperone (Lee et al., 2010; Yoon et al., 2016), could improve OIH by inhibiting ER stress. Recently, relations between opioids and ER stress have begun to step into the spotlight in some studies, though the data reported are somehow paradoxical. It is reported that morphine prevents spinal cord astrocytes from apoptosis induced by glutamate via reducing ER stress (Zhang et al., 2016). Low-dose morphine shows neuroprotection in Parkinson’s disease models through alleviating ER stress (Wang et al., 2018). On the contrary, morphine inhibits mitochondria damage-induced accumulation of PTEN-induced putative kinase 1 (PINK1) and consequently results in the inhibition of mitophagy and robust ROS production (Kong et al., 2019). And, exposure of hippocampal neurons to morphine leads to the imbalance between excitatory and inhibitory synapses, with up-regulation of intracellular ROS and sequential induction of ER stress (Cai et al., 2016). Furthermore, recently, a unique phenomenon was discovered that ER stress in the spinal cord was involved in the development of morphine tolerance (Qu et al., 2017). In our study, we not only confirm ER stress induction in spinal neurons after chronic administration of morphine, but also illustrate how ER stress is induced. We have previously demonstrated, for the first time, that morphine could induce the release of HSP70 from neurons via MOR/AKT/K_ATP_/ERK pathway (Qu et al., 2017). Here, we further found that a decline of intracellular HSP70, which was caused by morphine-induced efflux of this molecule chaperone, failed to maintain the homeostasis of ER. When the intracellular level of HSP70 was rescued by pharmacological and molecular methods, the ER stress caused by morphine could be alleviated. In addition, HSP70 was important not only for ER function, but also for neuroinflammation. Extracellular HSP70 primes NLRP3 inflammasome in microglia by binding with toll-like receptor 4 (TLR4) (Qu et al., 2017), which may be another cellular mechanism for OIH. Therefore, the best strategy to rescue OIH may be preventing the decline of intracellular HSP70 and the increase of extracellular HSP70 at the same time. Therefore, blockage of HSP70 release would be more effective than merely increasing neuronal HSP70. Glibenclamide (2 μg/10 μL, *i.t.*), a classic K_ATP_ channel blocker and a safe drug used clinically, could increase the tail flick latency by 64.7% (Figure 8B), while the rescue of HSP70 by adenovirus-mediated overexpression in neurons increased the tail flick latency by 42.5% comparing with that in the morphine-treated group (Figure 9B).

Previous studies have shown that ER stress-induced NMDAR activation is involved in many diseases of the central nervous system. Researchers found that amyloid-beta oligomers (AβO), the species implicated in synaptic loss during the initial stage of Alzheimer’s disease, induce ER stress in cultured neurons with an activation of NAMDR receptors (Costa et al., 2012; Halloran et al., 2013). ER stress and consequent NMDAR activation were also involved in other neurodegenerative diseases including Parkinson’s disease, amyotrophic lateral sclerosis (ALS), Creutzfeldt-Jakob disease (CJD), and Huntington’s disease (HD) (Halloran et al., 2013). Though NMDA receptors, especially the function of NR1 subunit, was important in the development of OIH (Anderson et al., 2015; Stoicea et al., 2015), the mechanism of ER stress to induce NMDAR activation remains elusive after morphine treatment. It was reported that deficiency of IL-10 led to the induction of ER stress, which caused expression of BiP and redistribution of SigR1 between components of ER and plasma membrane, and subsequently changed the specific organization of NMDAR (Koriauli et al., 2015). However, at present, we firstly report that morphine-induced ER stress activates PKA signaling, while inhibition of ER stress prevents phosphorylation of PKA and correspondingly suppress the phosphorylation of NR1 Ser897 induced by morphine (Figure 3). It was reported that activated PKA could phosphorylate NR1 at Ser897, and the inhibition of PKA with 6–22 amide could preserve morphine antinociception (Rodríguez-Muñoz et al., 2012). The diminished antinociceptive capacity of morphine caused by NMDA phosphorylation could only be improved by the inhibition of PKA, not by PKC and CaMKII (Rodríguez-Muñoz et al., 2012). These data, accompanied with our results, indicate that the evoked ER stress leads to the activation of NMDA receptor via PKA phosphorylation.

**FIGURE 10:**
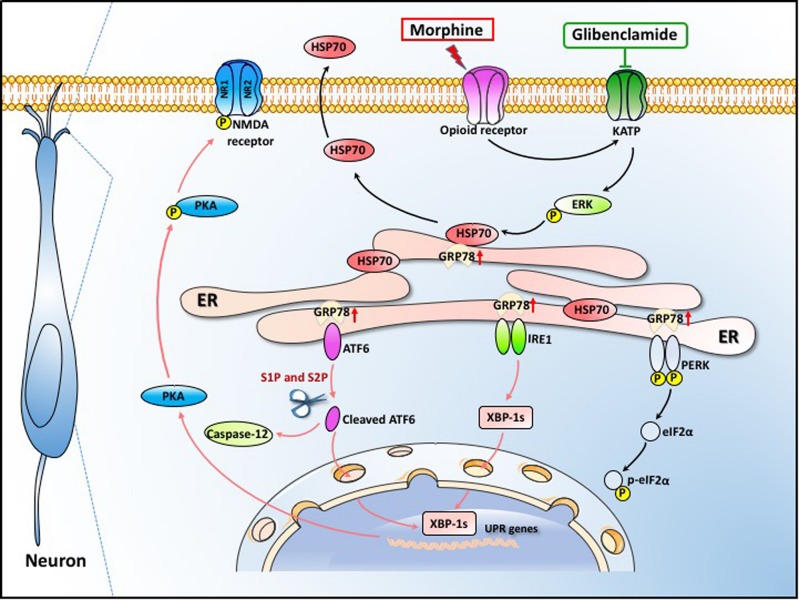
Schematic model indicates that glibenclamide alleviates OIH by inhibiting HSP70 release and ER stress. Morphine induces ER stress by the efflux of HSP70. The secretion of HSP70 is under the control of MOR/K_ATP_/ERK signal pathway. The efflux of HSP70 leads to the separation of GRP78 from IRE1α, ATF6 and PERK. ER stress enhances PKA-mediated phosphorylation of NMDA receptor which consequently leads to OIH. Glibenclamide as a K_ATP_ channel blocker inhibits the release of HSP70 induced by morphine, consequently alleviating OIH in rodents.

In summary, our data demonstrate that ER stress in the spinal cord neurons, mainly mediated by IRE1α and ATF6 pathway, is important in the development of OIH. Morphine-induced ER stress enhances PKA-mediated phosphorylation of NMDA receptor which consequently results in OIH. The efflux of HSP70 from spinal neurons induced by morphine via K_ATP_/ERK pathway was the primary cause of ER stress. Moreover, our study provides a clinically safe and effective drug, glibenclamide, to inhibit HSP70 release by blocking K_ATP_ channel and to effectively alleviate OIH in rodents (Figure 10). Our findings may represent a brilliant prospect for the improvement of OIH with glibenclamide and lay the groundwork for the treatment of patients suffering from chronic pain.

## Data Availability Statement

All datasets generated for this study are included in the article/Supplementary Material.

## Ethics Statement

The animal study was reviewed and approved by Nanjing Medical University Animal Care and Use Committee.

## Author Contributions

T-TL, JQ, and C-YW performed the experiments and analyzed the results. T-TL, XY, and LH carried out the animal experiments, cell cultures, and immunohistochemistry. FH, X-FW, and JQ carried out the Western blotting analysis. C-YJ, W-TL, and YH conceived of the study, participated in its design and coordination, and helped to draft the manuscript. All authors read and approved the final manuscript.

## Conflict of Interest

The authors declare that the research was conducted in the absence of any commercial or financial relationships that could be construed as a potential conflict of interest.

## Supplementary Material

FIGURE S1 The inhibition of HSP70 releasing by gliquidone suppresses morphine-induced ER stress and the phosphorylation of PKA and NR-1. Representative western blot images were shown in this figure. **(A)** Gliquidone inhibited the phosphorylation of ERK1/2 elevated by morphine. Gliquidone (200 μM) was given 15 min before morphine (200 μM, 1 h) administration. Cell extracts were collected and analyzed by western blot (*n* = 3). **(B)** Gliquidone administration (200 μM) 15 min prior to morphine (200 μM, 12 h) suppressed the morphine-induced HSP70 release in SH-SY5Y cells. Supernatants were collected 12 h after morphine treatment and determined by western blot (*n* = 3). **(C–F)** Pretreatment with gliquidone inhibited the up-regulation of ER stress molecule GRP78, IRE-1α, XBP1s and Caspase-12 induced by morphine in SH-SY5Y cells. Gliquidone (200 μM) was given 15 min before morphine (200 μM, 12 h) administration. Cells were collected 12 h after morphine treatment and analyzed by western blot (*n* = 3). **(G,H)** Gliquidone inhibited the phosphorylation of PKA and NR-1 induced by morphine in SH-SY5Y cells. Gliquidone (200 μM) was given 15 min before morphine (200 μM, 12 h) administration. Cells were collected 12 h after morphine treatment and analyzed by western blot (*n* = 3). A-H data were analyzed by one-way ANOVA (^∗^*P* < 0.05, ^∗∗^*P* < 0.01, ^∗∗∗^*P* < 0.001 vs. control, ^#^*P* < 0.05 vs. morphine-treated group).

DATA SHEETS S1–S10 The original unprocessed images of all western blots.
