# Supplementary figures and images for: Rescue of HSP70 in Spinal Neurons Alleviates Opioids-Induced Hyperalgesia via the Suppression of Endoplasmic Reticulum Stress in Rodents
Source: Front Cell Dev Biol. 2020 May 12;8:269. doi: 10.3389/fcell.2020.00269 (PMC7243285; doi:10.3389/fcell.2020.00269)

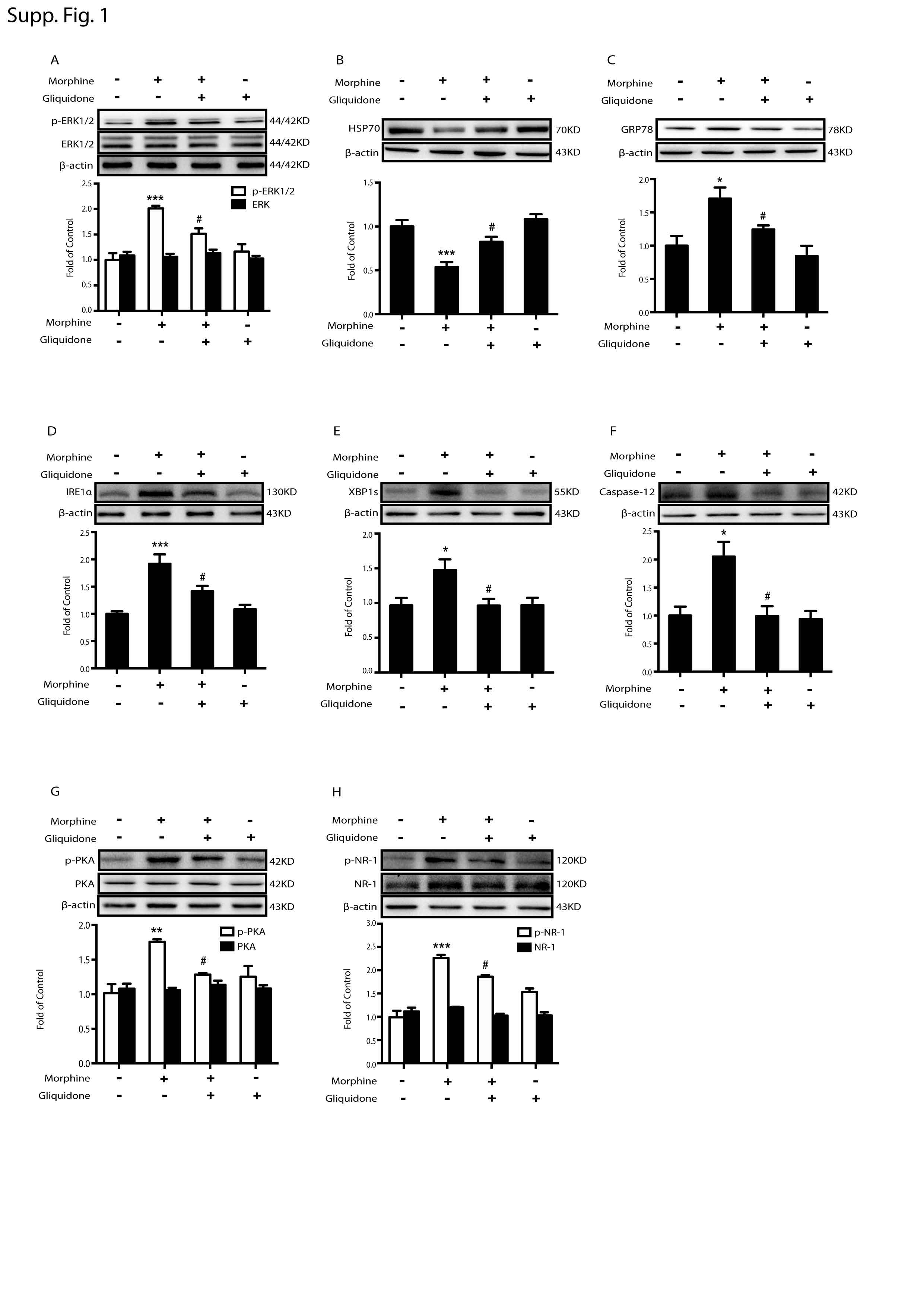

Supplement: FIGURE S1 — The inhibition of HSP70 releasing by gliquidone suppresses morphine-induced ER stress and the phosphorylation of PKA and NR-1. Representative western blot images were shown in this figure. (A) Gliquidone inhibited the phosphorylation of ERK1/2 elevated by morphine. Gliquidone (200 μM) was given 15 min before morphine (200 μM, 1 h) administration. Cell extracts were collected and analyzed by western blot (n = 3). (B) Gliquidone administration (200 μM) 15 min prior to morphine (200 μM, 12 h) suppressed the morphine-induced HSP70 release in SH-SY5Y cells. Supernatants were collected 12 h after morphine treatment and determined by western blot (n = 3). (C–F) Pretreatment with gliquidone inhibited the up-regulation of ER stress molecule GRP78, IRE-1α, XBP1s and Caspase-12 induced by morphine in SH-SY5Y cells. Gliquidone (200 μM) was given 15 min before morphine (200 μM, 12 h) administration. Cells were collected 12 h after morphine treatment and analyzed by western blot (n = 3). (G,H) Gliquidone inhibited the phosphorylation of PKA and NR-1 induced by morphine in SH-SY5Y cells. Gliquidone (200 μM) was given 15 min before morphine (200 μM, 12 h) administration. Cells were collected 12 h after morphine treatment and analyzed by western blot (n = 3). A-H data were analyzed by one-way ANOVA (∗P < 0.05, ∗∗P < 0.01, ∗∗∗P < 0.001 vs. control, #P < 0.05 vs. morphine-treated group). [file Image_1.JPEG]
